# A novel approach based on rough set theory for analyzing information disorder

**DOI:** 10.1007/s10489-022-04283-9

**Published:** 2022-12-01

**Authors:** Angelo Gaeta, Vincenzo Loia, Luigi Lomasto, Francesco Orciuoli

**Affiliations:** 1 Dipartimento di Scienze Aziendali - Management & Innovation Systems (DISA-MIS), Università degli Studi di Salerno, Via Giovanni Paolo II, 132, 84084 Fisciano Italy; 2 Ministero dell’Istruzione, ISS Manlio Rossi Doria, Via Manlio Rossi Doria Marigliano, Napoli, Italy

**Keywords:** Information disorder, Rough sets, Fuzzy rough sets

## Abstract

The paper presents and evaluates an approach based on Rough Set Theory, and some variants and extensions of this theory, to analyze phenomena related to Information Disorder. The main concepts and constructs of Rough Set Theory, such as lower and upper approximations of a target set, indiscernibility and neighborhood binary relations, are used to model and reason on groups of social media users and sets of information that circulate in the social media. Information theoretic measures, such as roughness and entropy, are used to evaluate two concepts, Complexity and Milestone, that have been borrowed by system theory and contextualized for Information Disorder. The novelty of the results presented in this paper relates to the adoption of Rough Set Theory constructs and operators in this new and unexplored field of investigation and, specifically, to model key elements of Information Disorder, such as the message and the interpreters, and reason on the evolutionary dynamics of these elements. The added value of using these measures is an increase in the ability to interpret the effects of Information Disorder, due to the circulation of news, as the ratio between the cardinality of lower and upper approximations of a Rough Set, cardinality variations of parts, increase in their fragmentation or cohesion. Such improved interpretative ability can be beneficial to social media analysts and providers. Four algorithms based on Rough Set Theory and some variants or extensions are used to evaluate the results in a case study built with real data used to contrast disinformation for COVID-19. The achieved results allow to understand the superiority of the approaches based on Fuzzy Rough Sets for the interpretation of our phenomenon.

## Introduction

The term *Information Disorder* [51] is used to address three phenomena related to distorting information-related processes: mis-information, dis-information and mal-information. In particular, *Information Disorder* is replacing the term *Fake News* along the discussions about the pollution related to the information diffusion. In fact, fake news represent only one of the different shapes of a whole complex phenomenon but it is an inadequate term to describe such phenomenon. More in detail, mis-information is when false information is shared but no harm is meant, dis-information is when false information is knowingly shared to cause harm, mal-information is when genuine information is shared to cause harm (often by moving information designed to stay private into public sphere). In such a context, it is possible to collocate fake news under the umbrella of mis-information or dis-information. Moreover, *propaganda* is a domain-specific term adopted to indicate true or false information spread to persuade an audience, but often has a political connotation. In order to clearly depict the criticality of information disorder, it is possible to cite WHO (World Health Organization) that introduced the term *infodemic* to indicate huge amounts of accurate or inaccurate health information that can lead to information disorder (mis-information, dis-information, mal-information and rumors) during a health emergency and can hamper an effective public health response.^1^ Infodemics also induce security threats. It is possible to note that new types of cybercrime may leverage both the information overflow and health-related panic [16].

Just with respect to fake news many initiatives have been launched. Firstly, fact checking services (mainly active on the Web) like, for instance, PolitiFact,^2^ FactCheck.org^3^ and Washington Post’s Fact Checker.^4^ According to this trend, Google makes available its aggregator of fact checked news^5^ and also Facebook provides its fact checker based on the work of independent fact checker organizations certified by ICFN.^6^ In particular the Facebook fact checking approach is based on five key phases: detection of possible fake news (selected through users’ feedback), content checking (verification of truth of facts and accuracy of information by means of consultation of sources), dis-information content marking (through labels visible for the users), decreasing the priority of dis-information content (putting such content after non-fake content in users’ visualizations), and limiting the information spreading capabilities of users who repeatedly share fake content. The authors of [5] argue that the effort undertaken by Facebook in regard to fact-checking, tagging, and flagging instances or appearances of fake news does not delete the problem but, on the contrary, it could feed such problem by convincing users that the unmarked (as fake) content shared through Facebook is real and, consequently, limiting their ability to decide. Therefore, the fight to fake news is limited because it does not consider all the aspects of information disorder. Starting from these considerations, it clearly emerges the need for approaches to monitor and analyze all the phenomena related to information disorder that have been better characterized by the authors of [51] who affirm that it is useful to consider three elements to understand such disorder: agent, message and interpreter. It is interesting to know more about these elements. With respect to the agents, it emerges the need to know about type (e.g., political parties, intelligence services or group of private citizens), organization (e.g., individual persons or groups), motivations (e.g., financial, political, social, etc.), intents (e.g., targets, intention to mislead or not, intention to harm or not) and technology support (e.g., use of automatic mechanisms) of them. With respect to the messages, it is interesting to investigate about the intended durability of the message, its accuracy, and so on. Lastly, with respect to the interpreters, they represent the audiences reached by the messages and they are rarely passive recipients of information, given that they interpret information and act accordingly to their own socio-cultural status, political positions and personal experiences.

This paper describes and evaluates a new computational approach to analyze information disorder based on Rough Set theory. The motivation to adopt this theory is that its constructs and operators are valid tools to model key elements of information disorder, such as the messages and the interpreters, and reason on the evolutionary dynamics of these elements. The approach aims to evaluate: *a)* the effects due to the circulation of new information in groups of social media users, i.e., the interpreters that are characterized with respect to attributes of interest of an analyst, and *b)* to comprehend these effects in the light of news characterizations, i.e., the messages that consider typical attributes of the information disorder discipline such as the nature and topic of information and user sentiment. The added value consists in an immediacy of the interpretation of the effects as variations of accuracy and complexity measures allowing to increase situational awareness of the analyst.

The main results described by this paper are: i) applying Rough Set theory for providing novel definitions of data-driven models of messages (shared news) and interpreters (social media users); and ii) applying measures based on roughness and entropy to characterize the evolution of disorder in the information spreading space.

The structure of the paper is as follows. Section 2 presents related works. Section 3 presents background information of Rough Sets and some variants and extensions adopted in the paper. Section 4 presents the method and Section 5 describes how it is implemented in some formal settings of Rough Set theory. Section 6 presents a case study, based on real data, defined to validate the results that are discussed and compared in Section 7. Section 8 draws conclusions and presents future works.

## Related works

The study of information disorders from the computational perspective follows two main directions. The first one is based on modelling and analysing information disorder by using complex networks. The second one is mainly based on Data Science and Artificial Intelligence [17], in particular,on machine learning and text mining methods [20]. Such simplification is adopted to understand also the different aims of researchers involved in such field of studies. Complex networks [22] are mainly used in order to comprehend more on both the spreading of low-credibility content and the motivations, decisions, intent of groups or individual online accounts sharing such content. The study of echo chambers, polarisation phenomenon, opinion dynamics, etc. can be faced through the aforementioned approaches. On the other side, machine learning and text mining [44] approaches are especially used to deal with disinformation and fake news detection or early detection. In this context, the selection of effective subsets of features is crucial for the efficacy of the proposed approached since the analysis of information disorder relies on the correct classification of content (e.g., real, fake) and users (i.e., interpreters) behaviors, and these are tasks that benefit from good attributes reduction and features selection algorithms. Approaches such as [29] and [48] are especially useful since rely on the Granular Computing paradigm for the tasks of attributes reduction and feature selection. These approaches based on Granular Computing have the advantage of considering different levels of granularity (multi-granularity) in the attribute reduction and feature selection for improving classification performances. Both the directions foresee the use of adequate datasets, typically collected from online social networks like Twitter, Facebook, Reddit, etc. and often integrated with other data coming from different sources, for carrying out the research activity. Collecting high-quality data in such a context is one of the main open challenges in this research field.

With respect to the first direction, a major boost to mis-information study has been provided by pandemics and vaccine campaigns. Some works, like [25] and [11], adopt epidemiological models to study mis-information, other works exploit analysis of social media content and network (see [52], [42] and [41]). Moreover, the recent covid-19 pandemic has inspired works based on both qualitative (see [33] and [15]) and quantitative approaches (see [7, 11, 46] and [21]). In particular, the authors of [21] analyzed covid-19 related conversations on the Italian Facebook by means of text analysis (empowered by linguistic and sentiment analysis), network analysis and statistics. Data are gathered from public posts (in Facebook) dealing with covid-19, i.e., where the text contains references to terms like *covid-19*, *coronavirus*, *sars-cov2*, etc. The focus was on controversial topics like *5G*, *labs* and *migrants*. Real and fake news are pre-classified by using the knowledge about their sources. Network structures like the diffusion network are constructed and analyzed. The numerical results of the aforementioned methodological phases are interpreted and discussed for obtaining a set of insights related to the emergence of a coordinated propaganda, polarization of groups, engagement of reliable and unreliable sources also with respect to specific topics, characteristics of accounts, influential accounts, peaks of positive and negative sentiments, clusters with a significant share of disinformation, etc. The same authors provided also a comparison between two social media platforms (Twitter and Facebook) [53] with respect to the diffusion of disinformation related to covid-19 by adopting the same tools. Another work [33], adopting network and socio-linguistic analysis, focused on characterizing mis-information online communities, in the context of covid-19, showing such communities are denser and more organized than informed communities, with a possibility of a high volume of the misinformation being part of disinformation campaigns. Other works focused on quantitative analysis to understand much more about fake news related to specific topics in a given context. Among these works, the authors of [36] provides quantitative results about fake news on covid-19 during the last months of 2019 and the first months of 2020.

Furthermore, with respect to the second direction, that is less relevant with respect this paper, the work [44] provides knowledge about fake news in social media and, in particular, it addresses both the characterization of fake news and the detection of them by defining a general data mining framework for feature extraction (see [8, 9, 32, 45], and [4]) and model construction (see [10, 47, 50], and [43]). More in detail, it is possible to recognize a new trend followed by papers proposing approaches based on machine learning and deep learning (see [3, 35] and [23, 26, 27, 31, 32, 40, 54] and [30]). Lastly, approaches based on sentiment analysis are described in [6] and [2].

With respect to the pertinent scientific literature, the proposed approach can be positioned among the works aiming at studying phenomena related to the diffusion of mis-information, dis-information and mal-information. The works on fake news detection are far, in terms of objectives, from the present work. Even if the last one can exploit the results of fake news detection methods and tools. However, the difference between the present work and the similar ones is essentially in the use of a completely different formal framework. In fact, there is no evidence, at the date of writing of this paper, that Rough Set Theory is adopted in the context of information disorder, despite one work (with different objectives) provided by the same authors [1].

## Background on rough set and fuzzy rough set theories

This section reports background information on Rough Set and Fuzzy Rough Set Theories. In particular, the section focuses on the methods adopted in this paper.

### Rough sets, probabilistic rough sets and neighborhood rough sets

Rough Sets (RS) have been introduced by Zdzislaw Pawlak [37] as an extension of set theory for the study of intelligent systems characterized by imprecise information. A rough set is a formal approximation of a conventional crisp set in terms of a pair of sets which give the lower and the upper approximations of the original set. A key concept of RS is the indiscernibility relation. This is a binary relation that express the fact two objects are indiscernible (or indistinguishable) on the basis of their descriptions. Let us define formally this concept.

An Information System is a pair *IS* = (*U*,*A*) where the non-empty finite set *U* is the universe of discourse and *A* is a non-empty finite set of attributes describing the objects *x* ∈ *U*, and *A* is such that: $a: U \rightarrow V_{a}$, where *V*_*a*_ is a set of values of attribute *a*, called domain of *a*. *IS* can be represented by a data table where rows are the objects and columns are the attributes. If *A* = *C* ∪ *D* where *C* is a non-empty set of condition attributes and *D* is a non-empty set of decision attributes, an Information System is called Decision System,

An indiscernibility relation *I*_*B*_ on *U*, where $B \subseteq A$, is defined as it follows:

1 $$ I_{B} = \{(x, y) : x, y \in U \wedge \forall b\in B, b(x) = b(y)\}. $$

Once defined *I*_*B*_ it is possible to create a partition of *U* where each part is an equivalence class based on *I*_*B*_:

2 $$ {[x]}_{B} = \{y \in U : (x, y) \in I_{B}\}. $$

All the equivalence classes (called also information granules) [*x*]_*B*_ form the blocks of the partition *U*/*B*. Changes to *B* lead to changes in the composition of granules.

Let $X \subseteq U$ a subset of the Universe, $B \subseteq A$ a subset of attributes and *I*_*B*_ an indiscernibility relation *I*_*B*_ on *U*, it is possible to define:

3 $$ \underline{B}(X) = \{x \in U : [x]_{B} \subseteq X\}, $$

4 $$ \overline{B}(X) = \{x \in U : [x]_{B} \cap X \neq \emptyset\}. $$

$\underline {B}(X)$ and $\overline {B}(X)$ are the *B-lower approximation* and the *B-upper approximation* of the set $X \subseteq U$.

Equations (3) and (4) are two approximations of the target set *X*. Equation (3) is also called *positive* region (POS) and consists of all the equivalence classes that are included in *X*. Equation (4) consists of all the equivalence classes which have non-empty intersection with the target set. In other words, (4) is the complete set of objects that are *possibly* members of *X*. Its complement, i.e. $U- \overline {B}(X)$ is also called *negative* region (NEG) and contains the set of objects that can be definitely ruled out as members of *X*.

The *B-boundary region* characterizing the vagueness of the concept (set) *X* is:

5 $$ BND_{B}(X) = \overline{B}(X) - \underline{B}(X). $$

More in detail, if *BND*_*B*_(*X*)≠*∅* the target set *X* is a Rough Set, otherwise *X* is crisp.

A limitation of traditional RS theory is that it does not allow any uncertainty in the definition of lower and upper approximations and, to introduce a certain degree of tolerance in the definition of the two approximations, the probability approximation space was brought into RS [56]. Probabilistic Rough Sets (PRS) use the conditional probability, $P(X|[x])=\frac {|X\cap [x]|}{|[x]|}$, to define the lower and upper approximations as follows:

6 $$ \underline{B}(X) = \{x \in U : P(X|[x]_{B}) \geq \alpha\}, $$

7 $$ \overline{B}(X) = \{x \in U : P(X|[x]_{B}) > \beta\}, $$

where *α* and *β* (0 ≤ *β* < *α* ≤ 1) are two thresholds and establish the tolerance degree used to determine both lower and upper approximations. A boundary region can be evaluated with (5).

In application based on social media, data formation can be a complex issue involving combination of discrete and continuous variables. In order to use RS or PRS data needs to be discretized by using a suitable approach (e.g., quantiles/percentiles, uniform distribution). However, to deal with continuous variables, it is possible to adopt a variant of traditional RS: the Neighborhood Rough Sets (NRS). The main difference is the replacement of the binary relation of indiscernibility with a binary neighborhood relation.

A neighborhood of an element is an information granule that contains the element and the elements that are close to it. [55] defines a neighborhood relation as follows. For each element of an Universe, *x* ∈ *U*, and given a distance function $D : U \times U \rightarrow R^{+}$, the neighborhood of *x* is defined as:

8 $$ n_{d}(x) = \lbrace y | D(x,y) \leq d \rbrace $$

where *d* ∈ *R*^+^ is a threshold value. Lower and upper approximations of a target set can be constructed using neighborhoods as parts in (3) and (4).

### Fuzzy rough sets and fuzzy variable precision rough sets

Another possibility to deal with continuous variable is to use variants of RS based on Fuzzy theory. The relation between Rough and Fuzzy Sets is discussed in [14] that proposes two ways to combine the two theories. One of the way presented, usually knows as Fuzzy Rough Sets (FRS), is to turn the crisp indiscernibility relation of (1) into a fuzzy indiscernibility relation.

Before describing how FRS models define lower and upper approximations, let us briefly introduce some fuzzy logic operators such as t-norm, implicator, and negator. Details can be found in [38].

A t-norm is a function $T:[0,1]^{2} \rightarrow [0,1]$ that is monotone, commutative, associative and satisfies the boundary condition, i.e., *T*(*x*,1) = *x*. Most common t-norms are the minimum, $T(x,y) = {\min \limits } \lbrace x, y \rbrace $, and Lukasiewicz, $T(x,y) = {\max \limits } \lbrace x+y-1, 0 \rbrace $.

An implicator is a function $I:[0,1]^{2} \rightarrow [0,1]$ that satisfies *I*(1,0) = 0, *I*(1,1) = *I*(0,1) = *I*(0,0) = 1 which is decreasing in the first and increasing in the second argument. If *I* satisfies *I*(1,*x*) = *x* for all *x* ∈ [0,1], it is called a border implicator.

A negator is a function $N:[0,1] \rightarrow [0,1]$ that is decreasing and satisfies *N*(0) = 1 and *N*(1) = 0. A negator is called involutive iff *N*(*N*(*x*)) = *x* for all *x* ∈ [0,1]. The standard negator is *N*(*x*) = 1 − *x*.

Let us consider the fuzzy tolerance relation on an attribute *a* proposed in [24]:^7^

9 $$ R_{a}(x,y) = 1 - \frac{|a(x)-a(y)|}{|a_{\max} - a_{\min}|} $$

and let $B \subseteq A$. The fuzzy B-indiscernibility relation can be defined as:

10 $$ R_{B}(x,y) = T (R_{a}(x,y)) $$

where *T* is a t-norm operator. In the context of FRS, lower and upper approximations can be generalized by means of an implicator *I* and a t-norm *T* [38]. The fuzzy B-lower and B-upper approximations of a fuzzy set $A \subseteq U$ are defined as:

11 $$ (R_{B} \downarrow A) (y) = {\inf}_{x \in U}I(R_{B}(x,y), A(y)) $$

12 $$ (R_{B} \uparrow A) (y) = {\sup}_{x \in U}T(R_{B}(x,y), A(y)) $$

The meaning of (11) and (12) is similar to the one of (3) and (4). Equation (11) is the set of elements necessarily satisfying the target concept *A* (i.e., the elements with strong membership) and (12) is the set of element possibly belonging to the target concept *A* (i.e., the elements with weak membership).

As the traditional RS, the FRS also have extensions and variants aimed at removing some weaknesses. FRS are sensitive to mis-classification and small variation of data [57] and, to address this issue, some extensions have been proposed such as the Fuzzy Variable Precisions Rough Set Model (FVPRS) that combines FRS and Variable Precision Rough Sets [58]. The FVPRS model proposed in [57] defines the lower and upper approximations on the basis of a parameter *α* as follows:

13 $$ \begin{array}{@{}rcl@{}} (R_{\alpha} \downarrow A) (y) &=& {\inf}_{A(y) \leq \alpha}I(R(x,y),\alpha) \\ && \wedge {\inf}_{A(y) > \alpha} I(R(x,y),A(y)) \end{array} $$

14 $$ \begin{array}{@{}rcl@{}} (R_{\alpha} \uparrow A) (y) &=& {\sup}_{A(y) \geq N(\alpha)} T(R(x,y),N(\alpha))\\ && \vee {\sup}_{A(y) < N(\alpha)}T(R(x,y),A(y)) \end{array} $$

where *T*, *I* and *N* are respectively a t-norn, an implicator and a negator. For *α* = 0, the FVPRS is the FRS model. The parameter *α* plays a similar role to that of the two thresholds in PRS. In the PRS, the two thresholds are such as to admit a tolerance in the correct classification of the lower and upper approximations. In FVPRS, the parameter *α* incorporates a controlled degree into the FRS approximation operators so that the undesirable effect of mis-classification can be weakened.

## Overview of the method

This section presents the method defined to detect information disorder in social media groups. The section starts with the introduction of the concepts defined by Modis [34] of Complexity and Milestone for closed systems,^8^ the contextualization of these concepts to groups of social media users and the adoption of these concepts to detect information disorder.

### Complexity and milestones: an information theoretic definition

The idea behind the method proposed in this section is to contextualize some measures for evaluating the complexity of systems to the evaluation of information disorder in groups of social media users. Specifically, Modis in [34] defines Complexity and Entropy in an information theoretic perspective as follows:

- Complexity (*C*) is defined as *the capacity to incorporate information at a given time (or a measure of how difficult it is to describe at a given time)*
- Entropy (*S*) is defined as *the information content (or a measure of the amount of disorder)*

The same scholar, for closed systems, considers the following relations between *C* and *S*:

15 $$ C = \frac{dS}{dt} $$

16 $$ S = \int C dt $$

where *t* refers to continuous time.

To forecast the complexity of a system, Modis introduces the concept of Milestone (*I*), that is a cluster of events that can perturb a system, and relates this concept to *C* as follows:

17 $$ {{\varDelta}} C_{i} = \frac{I}{{{\varDelta}} T_{i}} $$

where *ΔT*_*i*_ is the time window between the *i* and *i* + 1 milestones.

### Modeling users and news

**Fig. 1:**
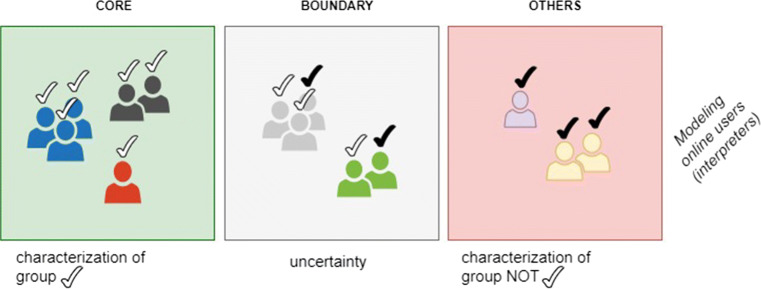
Social media users

To define *C* and *I* in the context of social media, it is necessary to model two main components of social media: users (that are the interpreters in Information Disorder terminology) and news (that is the message). Let us consider Fig. 1 showing a group of social media users.

From a graphical point of view, a group of social media users is divided in two parts: a CORE (the green square in the figure) and a BOUNDARY (the gray square in the figure). Inside each of these two parts, there can be different parts consisting of sub-groups of users who are similar or indistinguishable with respect to their behavior (e.g., social activities). These similar or indistinguishable parts are shown graphically with the same colors of users, e.g., all the blue users.

Let us suppose all the users can be classified with respect to a decision attribute such as their profession (for this attribute, a plausible value is, for instance, journalist). Assume that users classified as journalists are pointed out with a white tick in Fig. 1. In this case, the CORE of the group of journalists consists of the users that can be certainly classified as journalists, e.g., all users in the green square. The gray square represents a BOUNDARY between the CORE and the rest of the world identified as OTHERS in the red square of Fig. 1. The BOUNDARY consists of users for whom there is a degree of uncertainty related to their correct classification as journalists. This is because the sub-groups (users with the same color) with a mix of white and black ticks include users whose behavior is similar or indistinguishable but with different professions (i.e., classified differently). It is possible to note such aspect because in the gray square we have, for instance, two green users (same behavior) where the first one is a journalist and the second one is not a journalist. The red square, lastly, consists of all the other users that are classified with other professions - not journalists - and shown with a black tick.

The ability to correctly describe a group (e.g., journalists) at a given time *t*_0_, which is related to the definition of *C* reported above, depends on the size of the CORE and BOUNDARY areas and on the number of parts in which these two are divided. Intuitively, if the area of the square representing the CORE is large (i.e., there is a high number of users) and the number of parts is small, the group can be characterized quite precisely. Suppose that during a time window, [*t*_0_,*t*_1_], new information has circulated within a social media. This new information consists of content or news that have been circulated in the time period under analysis and corresponds to the milestone concept, namely *I*, mentioned above. Downstream of a milestone, the behavior of the users of the group under examination can change. This can lead to variations as regards both the areas under observation (CORE and BOUNDARY) and the number of parts in the areas. The extent of these variations provides information on the effect *I* has had in terms of information disorder.

Let us explain below how to characterize a milestone that consists of a set of news. This is an important step in our approach since a proper understanding of the measures depends on characterizing the milestone.

The approach is similar to that explained for the social media users. Milestone *I* consists of a set of contents (e.g., news) that can be classified with respect to some criteria, such as *topic* (e.g., gossip, terrorism), *nature* (e.g., fake or real, mis- or mal-information), and so on. Milestone *I* is described by means of a set of attributes, whose values are derived from the processing of comments and conversations (of users) associated to it. For instance, these attributes could report sentiment values extracted from users’ content (e.g., comments). Thus, for example, each news will be described by measures of sentiments extracted by the comments associated to it.

**Fig. 2:**
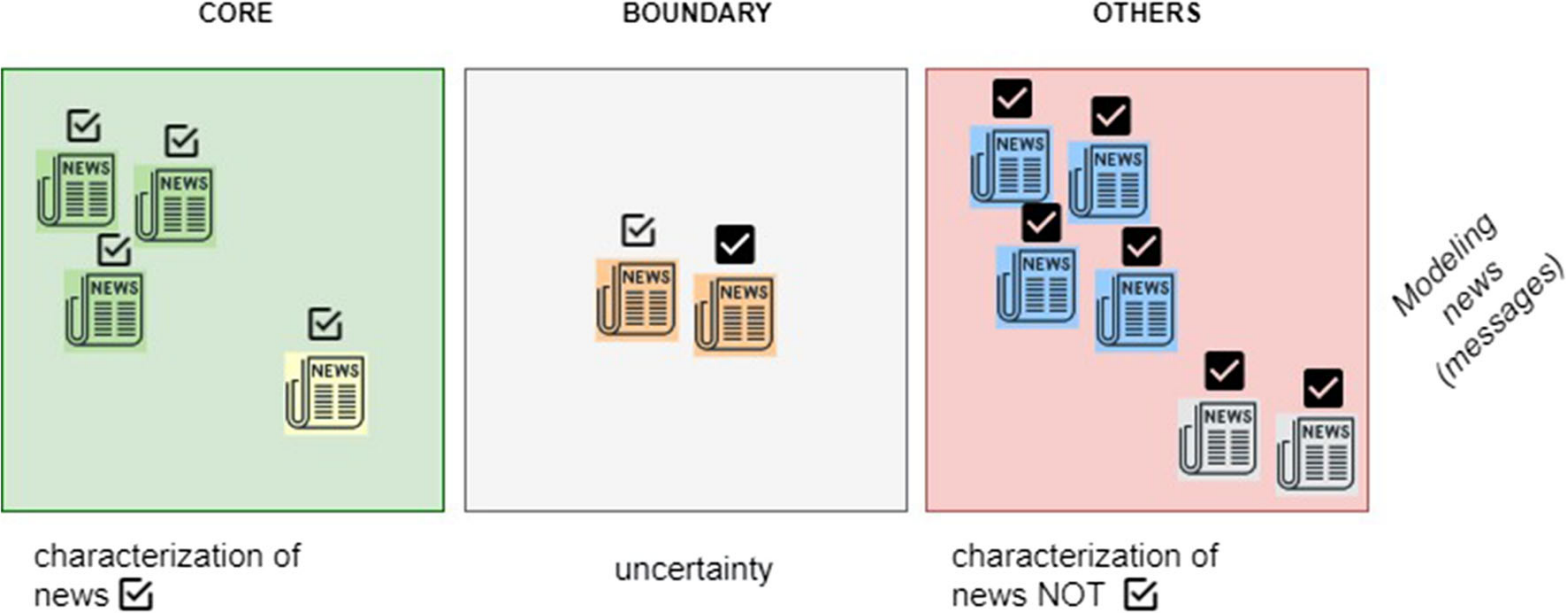
Milestone

Using these attributes, a characterization of *I* is graphically represented in Fig. 2 where, for simplicity, only one criterion is considered. In this case the criterion *nature* of the news, whose possible values are fake (white tick) or real (black tick), has to be considered and, in particular, the analysis is focused on studying the characterization of fake news. In Fig. 2, the green square is the CORE with respect to fake news, the gray square is the BOUNDARY with respect to fake news and, lastly, the red square is the rest of the world or OTHERS (e.g., real news). In a way similar to what has been done for social media groups, it is possible to identify parts consisting of news that are similar with respect to the sentiment expressed by the users. In Fig. 2, news associated to the same sentiment are represented by using the same color (e.g., green, blue, orange).

Of course, if more than one criteria is used to configure the target concepts (e.g., topic/nature and, in particular, gossip/fake news), further CORE and BOUNDARY regions will be considered. The meaning of a topic/nature CORE is as follows: parts of news correctly classified with respect to the topic/nature pairs that arouse very similar users’ sentiments. The size of the CORE represents, at a coarser granularity, the accuracy of the topic/nature classification of the milestone. On the other hand, at a finer granularity, the size of the CORE provides details on how many news, arousing similar users’ sentiment values, are correctly classified. This double level of information provided by a milestone helps to execute the overall analysis phase.

**Fig. 3:**
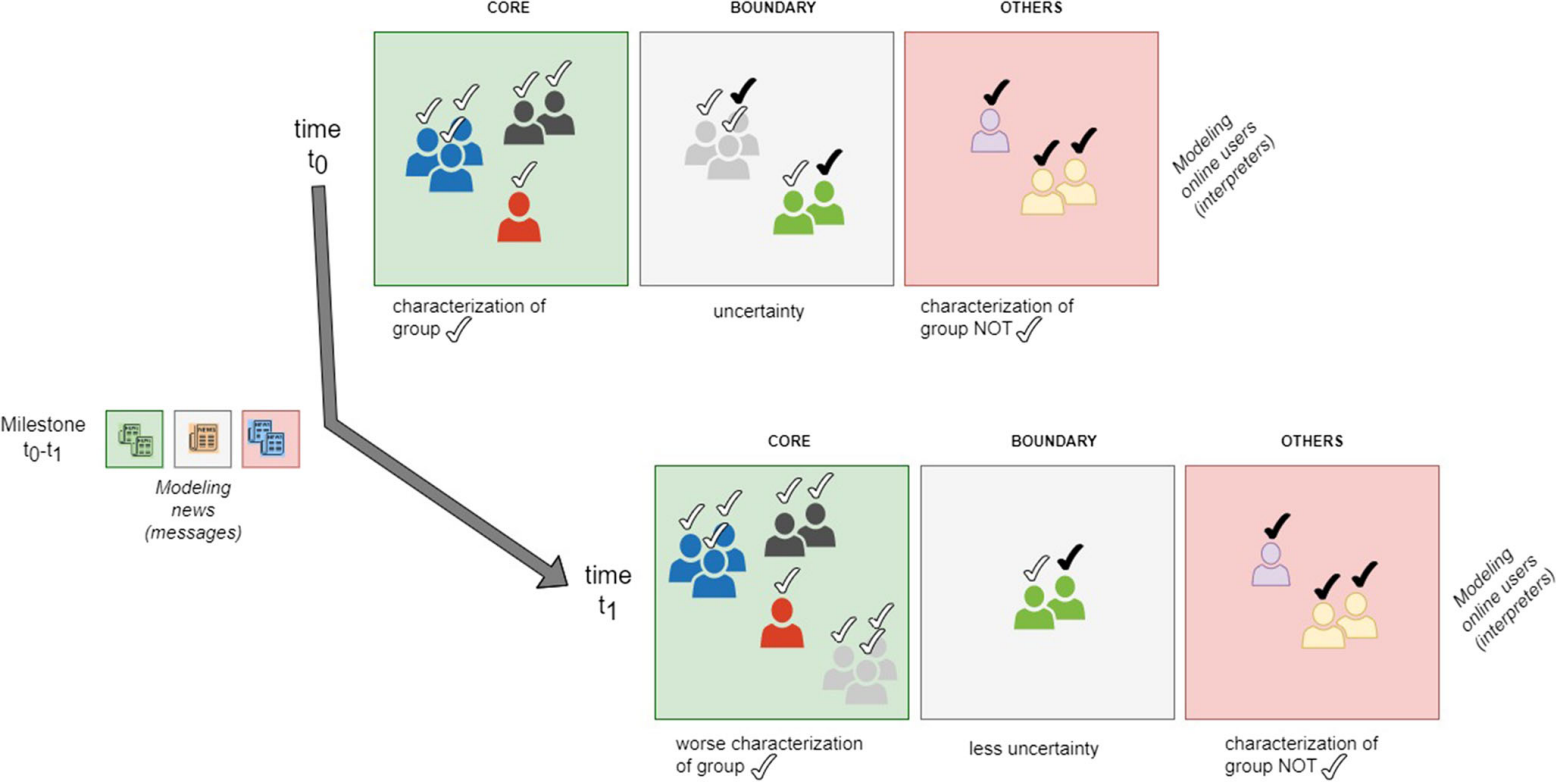
Analysis

An example of analysis that an analyst (or a social media provider) can execute with the aforementioned characterizations is described with the support of Fig. 3. At time *t*_0_, a group of users (target group) is characterized into its CORE and BOUNDARY regions. In the time window [*t*_0_,*t*_1_], new information (in terms of fresh news) is spread over the social media (e.g., Twitter) and the users create additional contents, such as textual comments, related to this information. Starting from this information and the content produced by the users, milestone *I* is characterized as described above. At time *t*_1_, the effect of milestone *I* in terms of information disorder can be analyzed by evaluating the increase or decrease of the CORE and BOUNDARY regions for the social media users as well as cohesion or fragmentation of sub-groups in these two regions. The analysis can be done at different levels of abstraction or granularity. Let us consider, for example, the case shown in Fig. 3.

Figure 3 shows a possible evolution where the CORE of the target group increases its size and number of parts. At the same time, the BOUNDARY of this group decreases its size and number of sub-groups. No variation is observed in the red square. At a coarser level of granularity, the evolution of CORE and BOUNDARY regions informs that the target group is more differentiable from the other groups (i.e., the OTHERS of Fig. 3). However, from Fig. 3, it is observed that the number of sub-groups in the CORE increased and the number of sub-groups in the BOUNDARY decreased. In this case, the overall increase of the CORE is balanced by a fragmentation of its sub-groups. Therefore, the ability to correctly differentiate the target group from the other groups is counterbalanced by a reduced ability to characterize its CORE. This may be positive or negative depending on the characterizations of the groups (of users) and milestone *I*.

At this level, in fact, the analysis results answer the following question: what is the influence that a set of news (with a specific accuracy degrees on the criteria topic/nature) has on the social behavior of a target group of user? For instance, what is the influence that a set of news, with 0.2 accuracy on gossip/real and 0.7 accuracy on gossip/fake, has on the journalists’ attitude of sharing fake or real content. In the light of the evolution shown in the Fig. 3, the answer to this question is that the effect is an increase of information disorder. The milestone *I*, in fact, had the effect of increasing the number of journalists (correctly classified) who share content more accurately characterized as gossip/fake.

At a finer granularity level, the analysis has the objective of providing details on the observed variations. By zooming into the CORE region, it is possible to observe the sub-groups within the CORE, where a social media provider could understand that fragmentation or cohesion of a target group are due to specific sentiments related to the news of milestone *I*.

Moreover, further types of variation, not shown in Fig. 3, could be observed. For instance, the size of the CORE may be reduced after the milestone. This means that the target group under analysis has a larger BOUNDARY area and it is more difficult to differentiate this group from the others. It is evident that this situation is not preferable as regards the monitoring of the information disorder in the groups since the ability to correctly discern the target group is a-priori reduced.

## Defining the method in rough set theory formal settings

In the this sections the RS concepts and operators are used to model a social media group of users and to define measures for *C* and *I*.

### Evaluating the complexity of a group of social media users

Let *U* be a set of on-line users of a social media, *A* = {*a*_1_,*a*_2_,...,*a*_*n*_} a subset of condition attributes, and *D* = {*d*} a decision attribute.

The condition attributes of *A* serve to characterize social media users. They can refer to information relating to online activities such as the number of shared contents. Values for *d* can be derived with a profiling function of interest. For instance, values for *d* can be related to users’ occupation, such as *journalist* or *scientist*, or behaviors such as *heather* or *troll*. The decision attribute *d* can be used to divide users into groups, or classes, and a social media analyst may be interested in evaluating the effects that new information circulating on social media can have on those classes.

Let *R* be a binary relation over *U* and let:

- {*U*/*D*} = {*TC*_1_,*TC*_2_,...,*TC*_*n*_} be a partition of *U* over *D* that represents the groups of users (e.g., journalist, scientist, and so on)
- {*U*/*A*} = {[*u*]_*R*_} be a partition of *U* over *A* that represents the users that are indistinguishable or similar (it depends from the specific nature of the binary relation *R*) with respect to condition attributes.

Let *TC*_*j*_ with *j* = 1,2,...,*n* be a target group of users, the complexity of this group at time *t*_0_ can be evaluated as follow:

18 $$ C_{t_{0}} (TC_{j}) = <Def(TC_{j}), IC(TC_{j}), EC(TC_{j})> $$

where *Def*(*TC*_*j*_) is a degree of definability of the group *TC*_*j*_, *IC*(*TC*_*j*_) and *EC*(*TC*_*j*_) are, respectively, degrees of internal and external cohesion of the group *TC*_*j*_. To simplify the reading, the subscript relating to time has been omitted.

*Def* informs on how much is difficult to define a group and is measured in relation to the degree of accuracy of prediction of a class of users. An high degree of accuracy allows to clearly describe a class. It also appears reasonable to hypothesize that if information causes disorder within a group of users this is reflected in a different degree of classification accuracy. *IC* and *EC* inform how much the users of the CORE and BOUNDARY regions are cohesive in terms of equivalence or similarity of behavior. These indicators are evaluated with entropy measures.

With reference to Fig. 1, the target group *TC*_*j*_ is a Rough Set or a Fuzzy Rough Set depending on the specific binary relation. The green square (i.e., the CORE of the group) represents the lower approximation, or positive region of *TC*_*j*_. The correct evaluation of this approximation depends on the specific formal setting adopted (i.e., (3) for RS, (6) for PRS, (11) for FRS and (13) for FVPRS.)

The gray square (i.e., the BOUNDARY of the group) represents a boundary region. The evaluation of the boundary regions is always done with a set difference between the upper and the lower approximations (see, for the traditional RS, (5).

It is possible to define:

19 $$ Def(TC_{j}) = 1 - \frac{|Low(TC_{j})|}{|Upp(TC_{j})|} $$

with |.| a cardinality, *Low* and *Upp* are, respectively, the lower and upper approximations of the set *TC*_*j*_ obtained by using the formulas for the different formal settings described in Section 3 (i.e., (3) and (4) for RS, (6) and (7) for PRS, (11) and (12) for FRS, (13) and (14) for FVPRS). If *TC*_*j*_ is crisp, naturally *Def* = 0 indicating that the group can be precisely described. As mentioned, *Def* refers to a difficulty in describing the group of users in terms of a partition of users (e.g., equivalence classes of users with the same behavior). A roughness measure (1 − *accuracy*) fits for this purpose since it represents the degree of incompleteness of knowledge about a group of users.

A measure of entropy for partitions, such as the one reported in [13], can be used to evaluate *IC* and *EC* in the case of a crisp indiscernibility relation (i.e., RS and PRS cases):

20 $$ IC = - \sum\limits_{i=1}^{n} \frac{|p_{i}(Low)|}{|Low(TC_{j})|}\log\frac{|p_{i}(Low)|}{|Low(TC_{j})|} $$

21 $$ \begin{array}{@{}rcl@{}} EC &=& - \sum\limits_{i=1}^{m} \frac{|p_{i}(Bnd)|}{|Low(TC_{j}) - Upp(TC_{j})|}\\ && \log\frac{|p_{i}(BND)|}{|Low(TC_{j}) - Upp(TC_{j})|} \end{array} $$

where *p*_*i*_(*Low*) and *p*_*i*_(*Bnd*) are the parts (i.e., elementary granules) belonging respectively to the lower approximation and boundary region. For a fuzzy indiscernibility relation (i.e., FRS and FVPRS cases), the non probabilistic entropy measure of a fuzzy set defined in [12] is used to evaluate *IC* and *EC*:

22 $$ H_{A} = - K \sum\limits_{i=1}^{n} (\mu_{i}\log\mu_{i} + (1-\mu_{i})\log(1-\mu_{i})) $$

where *K* is a constant value, usually settled to $\frac {1}{n}$, *H*_*A*_ is the entropy of a fuzzy set *A* and *μ*_*i*_ is the membership value of the *i* − *th* element of the fuzzy set *A*. Therefore, in order to get a value for *IC*, we need to calculate $H_{Low(TC_{j})}$. Otherwise, if we need to get a value for *EC*, we need to calculate $H_{Bnd(TC_{j})}$ where *Bnd*(*TC*_*j*_) = *Upp*(*TC*_*j*_) − *Low*(*TC*_*j*_).

If these measures are 0 there is maximum cohesion (e.g., only one part covers the whole area).^9^ The maximum values are: $\log (n)$ for (20), $\log (m)$ for (21) and $-2 \times 0.5\log (0.5)$ for (22). The maximum values indicate higher fragmentation (representing uniform distribution of the parts for (20) and (21) and higher uncertainty on memberships *μ*_*i*_ = 0.5 for (22)).

### Evaluating the milestone

Let *I* be a set of news that have been commented and shared by the social media users. Let *J* = {*j*_1_,...,*j*_*n*_} be a condition attributes set and *K* = {*k*_1_,...,*k*_*m*_} a decision attributes set. *J* consists of attributes related to sentiment of the users, *K* consists of attributes useful to classify the news, such as their topics and nature.

Let us derive values for condition and decision attributes sets as follows. For each news *i* ∈ *I*:

23 $$ j_{p} = \bigtriangledown_{u \in U} f(i, u) $$

where ▽ is an aggregation operator and *f*(*i*,*u*) is a function devoted to evaluate sentiment values from content of an user *u* related the news *i*. Of course, different functions *f* have to be used for different types of attributes (e.g., opinions, polarity, emotions). For the decision attributes, values can be derived with the support of content analytic to classify information:

24 $$ d_{q} = g(i) $$

where *g* can be, for instance, a topic modeling analytic, a method to classify fake/real news, and so on. Also in this case, different functions *g* must be used for different types of classifications. It is evident that the quality of the results of functions *f* and *g* impacts on the quality of results of the whole proposed approach. Such functions can be implemented by using operations provided by existing software libraries such as those described in Section 6.2,

Let *D* be a distance function, *R* an indiscernibility relation and define:

- {*I*/*K*} = {[*i*]_*R*_} a partition of *I* based on the equivalence relation *R* over the decision attributes set *K*. An equivalence class [*i*]_*K*_ is a set of news classified with a combination of the values of the decision attributes set *K*. To clarify this point, let us suppose we have two decision attributes, i.e. *topics* and *nature*, that can take respectively 3 (e.g., *gossip*, *scientific*, *report*) and 2 (e.g., *fake*, *real*) values. In this example, there can be 6 different equivalence classes of news based on the unique combination of values, e.g., *gossip/fake*, *gossip/real*, *scientific/fake*, *scientific/real*, *report/fake*, *report/real*. In other terms [*i*]_1_, [*i*]_2_, $\dots $, [*i*]_*z*_ form the concept family we need to approximate.
- {*I*/*J*} = {*n*_*d*_(*i*)} a partition of *I* based on the ditance relation *D* over the condition attributes set *J*. *n*_*d*_(*i*) = {*y* ∈ *I*|*D*(*y*,*i*) ≤ *d*}, where *d* is a threshold, is a neighborhood of *i* and includes all the news that are similar to *i*. *n*_*d*_(*i*) is a set of news that are perceived as similar on the basis of the sentiment values of the users.

Let us observe that elements in {*I*/*K*} are sets of news that can be approximated using the neighbors of {*I*/*J*} as parts in (3) and (4). In this way, it is possible to obtain a lower approximation and a boundary region that correspond to the CORE and BOUNDARY areas shown in Fig. 2. Therefore, a milestone referring to the set of news *I* can be described as a vector of accuracy values for each class:

25 $$ Mil(I) = <\frac{|Low([i]_{1})|}{|Upp([i]_{1})|}, ..., \frac{|Low([i]_{z})|}{|Upp([i]_{z})|}> $$

where *z* is the number of unique combination of the decision attributes. Each element of the vector gives information on how much news (that are perceived as similar on the basis of sentiment values) are properly classified with respect to a combination of decision attributes values.

### Detection of information disorder

In order to describe how to detect the information disorder led by a set of news, let us consider Fig 3 and limit to one group (g1) of social media users and two decision attributes: *topic* taking only one value (gossip) and *nature* taking two values (fake and real). In other terms, the family of concepts to approximate is {*gossip/fake*, *gossip/real*}.

The first step of the analysis is the evaluation at *t*_0_ of (18) to obtain the complexity of the group of users, i.e., $C(g1)_{t_{0}}$. At time *t*_1_, a milestone is evaluated with (25) and the complexity is reassessed with (18). Let us call $Mil(I)_{[t_{0}, t_{1}]}$ and $C(g1)_{t_{1}}$ these values. These steps are repeated for other time windows.

In general, a decrease of *Def* and *IC* suggests that the milestone led to a sharper separation among users’ groups and to a more cohesive core because the ratio between lower approximation and upper approximation grows. However, the meaning of these variations can be interpreted only with the milestone. Let us consider the following situation: < *ΔDef*_01_,*ΔIC*_01_,*ΔEC*_01_ >=< −,−,+ > meaning that there is a decrease of *Def* and *IC* and a increase of *EC* in the time window [*t*_0_,*t*_1_].

Assuming that the milestone is:

26 $$ \begin{array}{@{}rcl@{}} Mil(I)_{[t_{0}, t_{1}]} &=& < \frac{|Low(gossip/fake)|}{|Upp(gossip/fake)|},\\ && \frac{|Low(gossip/real)|}{|Upp(gossip/real)|}>, \end{array} $$

then we need to consider two cases: i) the first value is greater than the second one, and ii) the second value is greater than the first one. In the first case, the better characterization of fake news, with respect to real news, leading on a greater cohesion and a better characterization of users’ groups, means that information disorder increases. In the second case, the better characterization of real news, with respect to fake news, leading to cohesion and better characterization of users’ groups, means that information disorder decreases.

## A case study

The case study relates to an oppositional information disorder phenomenon [51] that is due to the fact that the interpreters of a message do not agree on the way it is encoded and therefore decline it in a different way and/or stop sharing and promoting it.

The scenario simulated in the case study is the following: a group of Twitter users comments and shares a set of real and fake news. At the time *t*_0_, the effect of this information is to characterize the users with respect to their behavior in terms of number of fake or real content commented and shared and, therefore, there are two subgroups of which we can describe the complexity with (18). This represents the initial state from which to start the analysis that relates to two consecutive temporal windows of similar width:

- [*t*_0_,*t*_1_], during which a similar number of real and fake news circulate,
- [*t*_1_,*t*_2_], during which a majority of real news circulate.

The objective of the case study is to verify whether, during the last time window, the reduced circulation of fake news implies effects that can be interpreted by means of the defined measures.

**Fig. 4:**
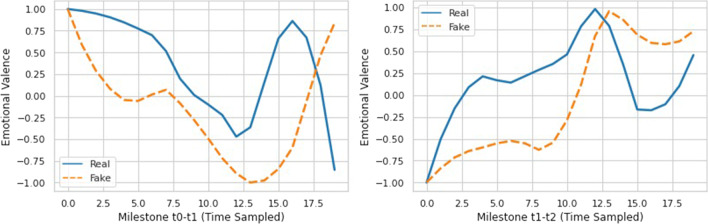
Emotional valence of the two milestones

It is clear that we are not only interested in the quantitative data (i.e., the number of fake and real news) but in verifying the effect of qualitative elements linked to the mass of information circulating in the aforementioned time windows. This, in essence, is what happens with the characterization of the two milestones (built in terms of sentiments of users). Furthermore, the emotional valence of the content^10^ (related to real and fake news) generated by the users during the two time windows has been calculated^11^ and shown in Fig. 4, where it is observed that in the first time window the trends of emotional valence, for real and fake, are very similar and start with positive values that tend to decrease, mainly for fake news, and then return to grow. For the second time window, a situation of similar trends can also be observed. However, in this case, while real news achieve a fair level of emotional valence for most of the time window, the emotional valence for fake news remain negative for a long time.

The experimentation carried out in this scenario has the following objective: to verify whether our results are capable of identifying the onset of information disorder phenomena. In the case study presented, the strong negative emotional valence of fake news in [*t*_1_,*t*_2_] is expected to lead some users to change their behavior (in terms of sharing real and fake news) due to an opposing reaction.

### Data gathering

The dataset has been constructed using the corpus provided by the Kaggle platform to help researchers to identify fake contents and to fight COVID-19 health misinformation.^12^ The original dataset is composed of thirty-five CSV files of both real and fake content shared on Twitter on covid-19 topic. The dataset for the experiment has been constructed by filtering and adjusting the original data. In particular: i) only news have been considered (claims have been dropped), ii) all news without publication date have been removed because it was not possible to put them in a temporal order as required by our analysis, iii) only news shared by at least three users and users who shared at least three news items have been included. Lastly, the number of fake and real news has been balanced. Lastly, for each different news in the dataset, Twitter APIs^13^ were invoked to extract the data related to comment related such news shared (on Twitter) by the considered users.

### Data wrangling

After the gathering phase, the news (in the collected data) have been divided in three different sets, one for each time window, basing on news publication month. Such sets have been used to construct three decision tables for the users, one for each considered time instant (*t*_0_, *t*_1_ and *t*_2_) and two decision tables for the milestones, one for each time window (*t*_0_-*t*_1_ and *t*_1_-*t*_2_).

In order to explain the compositions of the two types of decision tables (for users and for milestones) it is possible to consider Tables 1 and 2 respectively. In the former type, the universe is the set of users (one for each row). Note that we have a stable universe of 295 users along all the decision tables for the three time instants. In particular, each user is described by two condition attributes, namely nFake and nReal, reporting the number of fake news and real news shared by that user respectively. Moreover, there is a decision attribute, namely d, reporting 1 if that user shared more fake news than real news and 0 otherwise.

**Table 1:** Decision table of the users

	nFake	nReal	d
U0	3	0	1
U1	0	2	0
U2	0	5	0
...	...	...	...
Un	4	1	1

**Table 2:** Decision table of the milestone

	pol	sub	d
I0	0.456	0.345	Real
I1	0.122	0.211	Fake
I2	− 0.234	0.341	Fake
...	...	...	...
Im	0.3	0.123	Real

The latter type of decision table is based on the universe of news. Such universe is not stable along the two time windows. In fact, the idea is to evaluate the approach against two different milestones. In general, the rows for such decision tables represent different news and the columns describe such news. Two condition attribute are considered, pol (polarity) and sub (subjectivity), and one decision attribute, namely d, reporting if the news is *real* or *fake*. Polarity and subjectivity attributes provide average values, in the sense that each news is linked to a mean polarity value obtained by averaging all the polarity values (in the range [− 1, 1]) of text comments associated to the shares of such news. The same approach is used to calculate the mean subjectivity values. Note that the subjectivity of a text comment, associated to a share of a news, is a value in the range [0,1]. Polarity and Subjectivity values have been extracted by means of Spacy^14^ and Textblob.^15^

### Data analysis

The analysis has been done as follows:

- at *t*_0_, the Complexity of the two groups is evaluated with (18) starting from a decision table such as Table 1,
- at *t*_1_ the decision table of the users is updated on the basis of the new information circulated in [*t*_0_,*t*_1_] and a decision table of the milestone such as Table 2 is constructed. Starting from these tables, the Complexity of the groups is evaluated again with (18) and the milestone is characterized with (25).
- at *t*_2_, the same operations of the previous point are executed.

In this case study, the total number of users for the analysis is 295. Before *t*_0_, the total number of news is 105 divided in 77 real and 28 fake. In the time window [*t*_0_,*t*_1_], the total number of news circulated is 14 divided in 6 real e 8 fake and, lastly, in the time window [*t*_1_,*t*_2_], the total number of news circulated is 32 divided in 26 real e 6 fake.

## Experimental setting and results

The Complexity measure defined in the paper has been implemented and evaluated in four formal settings of Rough Set theory: traditional RS using 4 and 6 levels of discretization of condition attributes, PRS using 6 levels of discretization of condition attributes and with thresholds values *α* = 0.7 and *β* = 0.3, FRS and FVPRS with parameter values *α* = 0.03 and *α* = 0.05 (see Section 3).

The algorithms of the RoughSet package [39] of R^16^ has been used in all the cases but PRS where authors have developed the conditional probability measure for the evaluation of lower and upper approximations.

The algorithms of the package have been used to construct the decision tables of the users (e.g., Table 1), discretize the values of the conditional attributes of these tables obtaining 4 or 6 levels for nFake and nReal (only for RS and PRS), and to obtain the upper and lower approximations and the boundary region used to evaluate *Def*, *IC* and *EC* as defined in Section 5.1.

For the characterization of the milestones, a neighborhood relation has been used to evaluate the parts used in (25). A value of 0.09 has been used as threshold to define the neighbors. In this case, a Python implementation of the authors has been used to construct the decision tables of the milestones (e.g., Table 2 where pol (polarity) and sub (subjectivity) have been derived as mentioned in the previous section) and to obtain the upper and lower approximations in (25).

The results are as follows. The characterization of the milestones in the two time windows is reported in Table 3. Only the topic COVID is considered.

**Table 3:** Milestones

Time window	Real	Fake
t0-t1	0,36	0,36
t1-t2	0,25	0,00

**Fig. 5:**
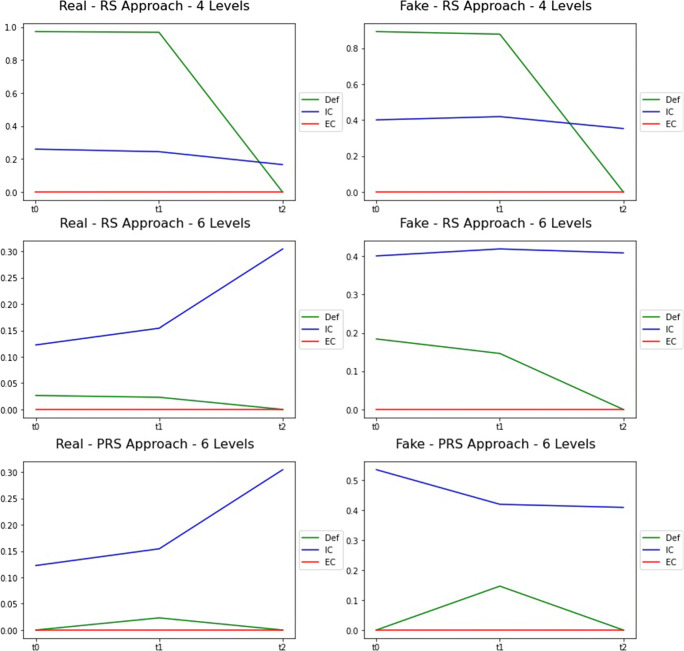
Results of RS and PRS

The values of Complexity for RS and PRS are reported in Table 4 and shown in Fig. 5.

**Table 4:** Results of RS and PRS

		RS4L			RS6L			PRS6L (0.3-0.7)		
Group	Time	Def	IC	EC	Def	IC	EC	Def	IC	EC
Real	t0	0,973	0,260	0,000	0,027	0,122	0,000	0,000	0,122	0,000
	t1	0,969	0,244	0,000	0,023	0,154	0,000	0,023	0,154	0,000
	t2	0,000	0,244	0,000	0,000	0,304	0,000	0,000	0,304	0,000
	t0	0,892	0,401	0,000	0,184	0,401	0,000	0,000	0,535	0,000
Fake	t1	0,878	0,419	0,000	0,146	0,419	0,000	0,146	0,419	0,000
	t2	0,000	0,353	0,000	0,000	0,409	0,000	0,000	0,409	0,000

**Fig. 6:**
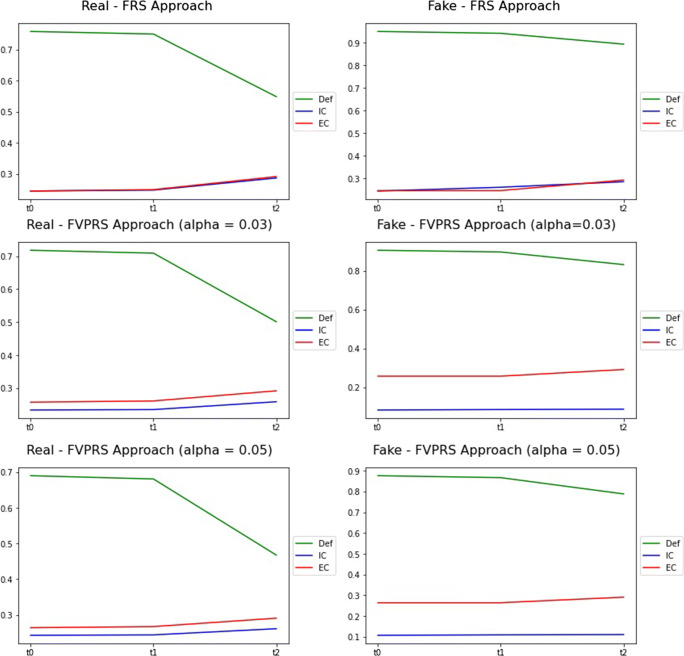
Results of FRS and FVPRS

The values of Complexity for FRS and FVPRS are reported in Table 5 and shown in Fig. 6.

**Table 5:** Results of FRS and FVPRS

		FRS			FVPRS (0.03)			FVPRS (0.05)		
Group	Time	Def	IC	EC	Def	IC	EC	Def	IC	EC
Real	t0	0,758	0,245	0,245	0,718	0,233	0,257	0,690	0,243	0,264
	t1	0,750	0,248	0,249	0,709	0,235	0,261	0,681	0,244	0,267
	t2	0,548	0,287	0,291	0,501	0,258	0,292	0,468	0,261	0,291
	t0	0,949	0,243	0,245	0,907	0,082	0,257	0,876	0,107	0,264
Fake	t1	0,941	0,260	0,245	0,898	0,085	0,257	0,867	0,109	0,264
	t2	0,893	0,284	0,291	0,832	0,087	0,292	0,788	0,111	0,291

### Discussion and comparison

This section discusses the results obtained and provides a comparison between the different algorithms used. First, let us discuss the characterization of the milestones in the two time windows reported in Table 3 as this is common to all the algorithms used.

In the time window, [*t*_0_,*t*_1_], the characterization of real and fake news in terms of polarity and subjectivity is the same. Looking at Fig. 4 (that, we recall, has been produced with a different approach based on the functionalities of *syuzhet* R package) this similarity is also confirmed by the similar trends of the emotional valence.

In the time window [*t*_1_,*t*_2_], the mass of information circulating is polarized towards real news (with 26 real against 6 fake news). However, from Fig. 4 a strong negative emotional valence for fake news can be observed and, from the second row of Table 3, that the fake news are not correctly classified with respect to the polarity and subjectivity of the users. This means that the strong negative connotation of emotional valence does not allow users to find a consensus with respect to the sentiment for such news.

**Fig. 7:**
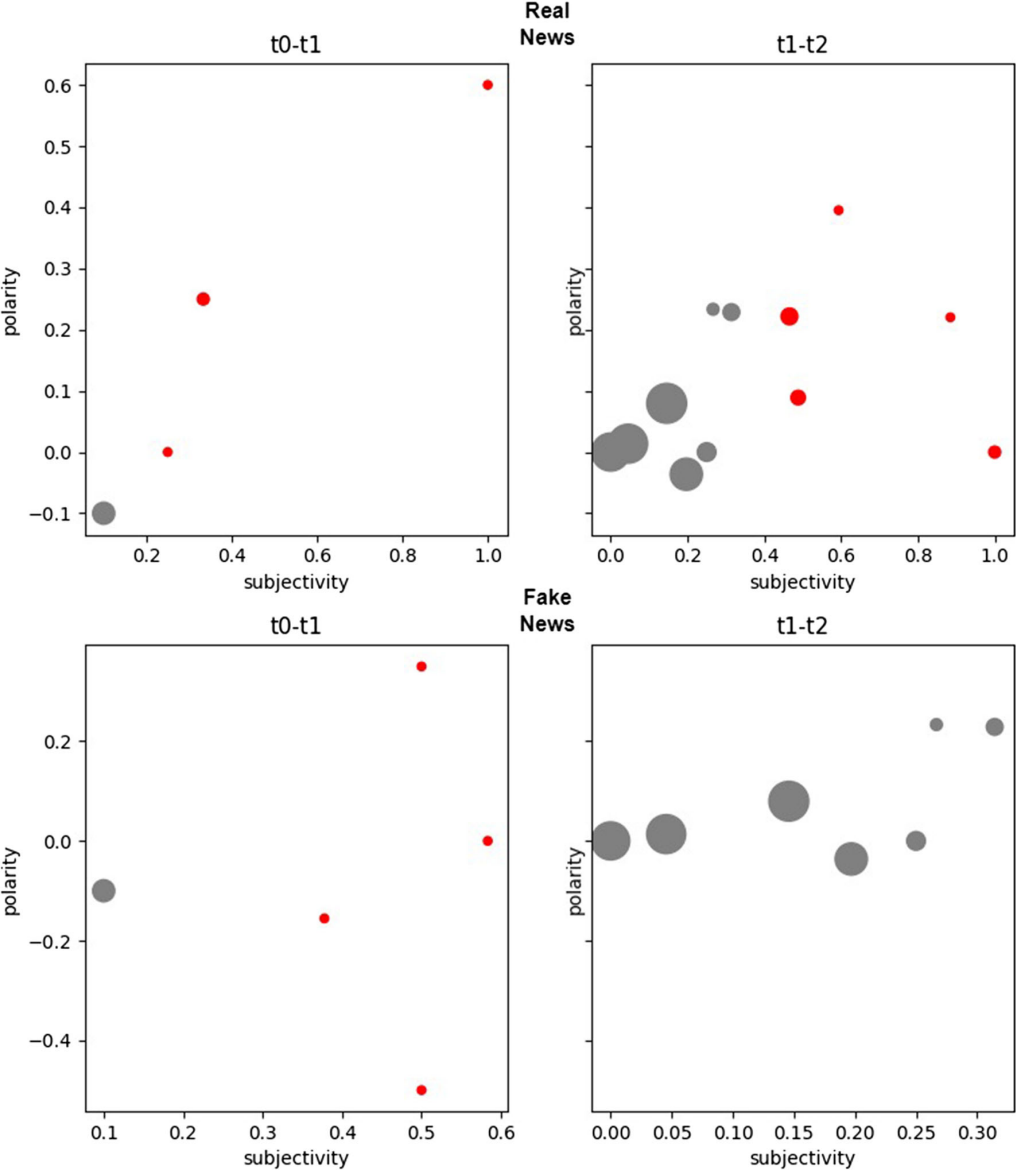
Evolution of the parts of the milestones

This can be observed with a fine grained analysis on the parts of the two milestones shown in Fig. 7. In Fig. 7, red circles represent the neighbors of the CORE (lower approximation) of fake and real news, gray circles represent the neighbors of the BOUNDARY (boundary region), and the size is proportionate to the cardinality of the parts. The disappearance of red circles for fake news in [*t*_1_,*t*_2_] indicates the aforementioned phenomenon: the strong negative emotional valence in this time window does not allow to find similarity of sentiment among users of social media.

It is in this time window that we expect our results to be able to identify the onset of information disorder phenomena linked to the strong negative emotional value of fake news that can result into an oppositional phenomenon.

Before moving on to the discussion of the results, it is useful to mention that the detection of the aforementioned behavior is an indicator for evaluating the validity of the results. In our case, in fact, it is not possible to evaluate the proposed approach by means of traditional indicators like precision, recall, AUC, and so on because there exists no ground truth to be used. The variance in terms of Complexity between the two time windows is compared to the trend of the emotional valence in order to provide a qualitative interpretation for the obtained results. Such interpretation is done by also taking into account the measures calculated for the news in the milestone reported in Table 3.

### Results of RS and PRS

With reference to Table 4, a first consideration deriving from the comparison of the RS4L and RS6L cases is that our results have a high sensitivity to the number of discretization levels with which the equivalence classes are defined. This is evident by observing the value of *Def* for both groups which, in the RS6L case, is much lower indicating that more equivalence classes enter the lower approximation. Looking at the value of *IC*, there is a reverse trend: quite stable for RS4L and growth for RS6L. This is due to the greater variety of equivalence classes that are created with the 6 levels of discretization. In the time window [*t*_0_,*t*_1_], however, the milestone does not have a noticeable impact in terms of behavior change. We recall that this first milestone is balanced in terms of number of fake and real news that circulated and the characterization of the milestone for real and fake news is the same.

**Fig. 8:**
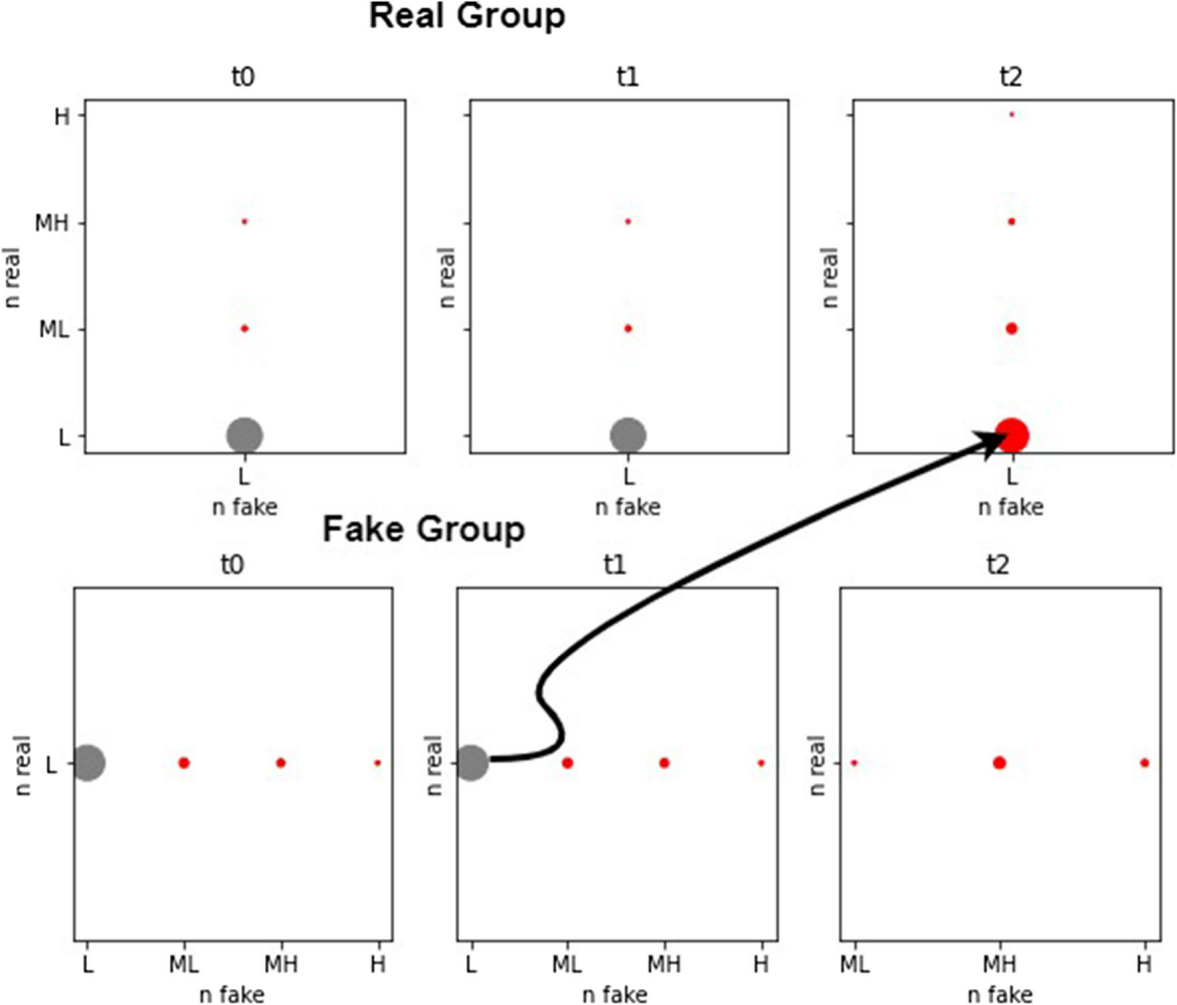
Evolution of the parts of the groups - RS4L

The situation in the time window [*t*_1_,*t*_2_] is different and allows us to verify the validity of the measures defined in the paper to detect phenomena of information disorder. In this period, the negative emotional valence of fake news led to a change of behavior of users who were part of the boundary region of the Fake Group and who stopped sharing fake news and become classified, at *t*_2_, in the CORE of the Real Group. This can be observed by analyzing the evolution of the parts of the groups in Fig. 8 that refers to the RS4L case. In Fig. 8 the labels *L*, *ML*, *MH* and *H* on the axes are the four levels obtained by discretizing the values of nFake and nReal attributes and indicate the quantity of news shared (Low, Medium to Low, Medium to High, High). With gray circles are shown the parts belonging to the BOUNDARY, and with red circles the parts that belong to the CORE. The size is proportionate to the cardinality of the parts. An arrow indicates the movement discussed.

A similar trend to that of Fig. 8 is also observed in the RS6L case with, obviously, the due differences in the cardinality of the boundary (much reduced in this last case). This movement has a beneficial effect on the ability to differentiate groups as seen from the *Def* values of Table 4 that become *Def* = 0 for both groups. The value *EC* = 0 has a different meaning in this time window. While in [*t*_0_,*t*_1_] it indicated the presence of a single part of users in the boundary regions, in this case it indicates the lack of parts in the boundary. In fact: *Def* = 0 means that |*Low*| = |*Upp*| and that |*Bnd*| = 0. In other words, the two groups of users are crisp sets.

Thus, in this time window, the parts of the boundary region of the Fake Group tend to change behavior (due to the negative emotional valence of the new fake news, these users stop to share fake news) and to aggregate to the lower region of the Real Group. This explains the increase of *IC* for the Real Group between *t*_1_ and *t*_2_ (which is more pronounced for the RS6L case) compared to a slight decrease of the *IC* for the Fake Group. By comparing RS6L and PRS6L, we can observe a similar situation between *t*_1_ and *t*_2_. However, with PRS6L we have introduced additional tolerance in accepting users as part of the lower regions. This has had an effect at *t*_0_ where lower and upper approximations are identical. From *t*_1_, the situation is the same as RS6.

### Results of FRS and FVPRS

The three algorithms show very similar results and trends (as can be observed from Table 5 and, more immediately, from Fig. 6). With FVPRS, the introduction of the parameter *α* allows to reduce the influence of mis-classification and small variation with the effect of slightly lowering *Def* for the two groups (a more pronounced decrease compared with FRS is achieved with *α* = 0.05) and has a reducing effect on the *IC* of the Fake Group. This means that the fuzzy equivalence classes in the lower region of the Fake Group are quite cohesive but numerically inferior to the parts of the upper region (as the *Def* values are about 0.8-0.9). Hence, the Fake Group has a very consistent boundary region which, however, appears very jagged as seen from the *EC* values. It is these users who, downstream of milestone [*t*_1_,*t*_2_], change their behavior.

**Fig. 9:**
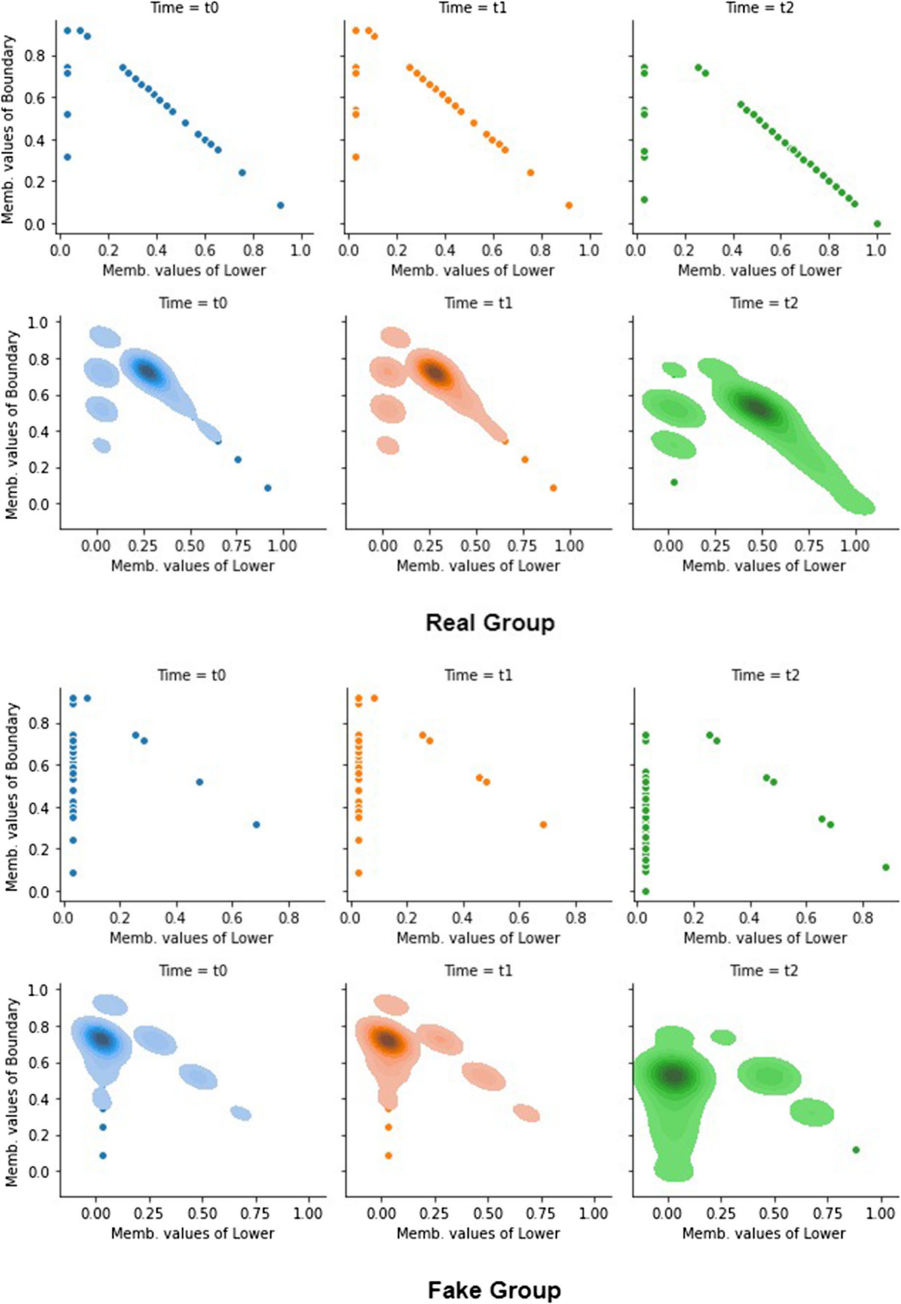
Evolution of the parts of the groups - FVPRS *α* = 0.03

For a finer granularity analysis, Fig. 9 is reported which is the equivalent of Fig. 8. Figure 9 shows a scatter plot where each point is an object of the universe and the *x* and *y* coordinates represent, respectively, the degree of membership of the object to the lower region and to the boundary region. To reveal the numerous overlaps, we have inserted a density graph (the lower part for each group of Fig. 9). Figure 9 refers to the case FVPRS with *α* = 0.03 but the situation is very similar for the other cases.

From Fig. 9, it can be seen that for the Real Group the most marked part of the density tends to move towards higher values of the membership degree of the lower region. This implies that the lower region increases its cardinality, and this fact is observed numerically with the values of *Def* in Table 5. Correspondingly, it is observed that for the Fake Group the most marked part of the density tends to shift towards lower values of the membership degree of the boundary region, resulting in a reduction in cardinality. Between *t*_1_ and *t*_2_, objects in the boundary region that decrease their cardinality do not substantially modify the lower region of the Fake Group and, therefore, impact the negative region of the Fake Group. But the negative region of the Fake Group is the Real Group and, therefore, this movement tends to reduce the Real Group’s *Def* value. The dynamic is, therefore, the one (already observed in the RS4L cases shown in Fig. 8) of users who pass from the boundary region of the Fake Group to the Real Group, modifying their behavior downstream of the milestone [*t*_1_,*t*_2_].

### Comparison of results

The adoption of the fuzzy indiscernibility relation, which models the behaviors of the users under analysis in a less marked way than the crisp indiscernibility relation, allowed to better analyze the relationship between the variations of *Def*, *IC* and *EC*. An immediately noticeable difference between the two types of RS theory algorithms (i.e., traditional and fuzzy) is related to the boundary region. In the experimentation carried out with the crisp indiscernibility relationship, the boundary region of the two groups had a single equivalence class between *t*_0_ and *t*_1_ that contained all the users (this fact can be observed from the *EC* value in such cases) that disappeared between *t*_1_ and *t*_2_. The PRS case is an exception as the tolerance introduced eliminates the boundary region at *t*_0_.

This complete emptying of the only block of users in the boundary region indicates that all these users change their behavior. This fact appears unrealistic and is caused by the difficulty of the crisp indiscernibility relation in modeling small variations in users’ behavior. With the adoption of a fuzzy indiscernibility relation, the situation appears more realistic and it is observed that the boundary region for both groups appears more disordered (i.e., different behaviors are considered) but constantly tends to reduce its cardinality without, however, reducing to 0. This means that the approaches based on FRS and FVPRS allow us to understand how only a part (and not all) of the users related to the boundary region of the Fake Group have changed behavior downstream of the milestone [*t*_1_,*t*_2_].

## Conclusions

The paper describes and evaluates in a case study based on real data some measures based on Rough Sets theory for the detection of information disorder phenomena. The results differ from the main computational trends and approaches to the study of information disorder as they focus on the possibility of interpreting the effects (such as the ability of groups to be identified and their level of cohesion) due to the circulation of new information on groups of social media users. This can be of considerable benefit to analysts and social media providers in analyzing information disorder and, possibly, in making predictions about its evolution in light of the characterization of milestones and social user groups.

The experimentation was carried out in a case study based on real data and related to oppositional information disorder phenomena. Four algorithms based on the Rough Set theory and its variants and extensions were tested. The analysis and comparison of results allowed us to understand the superiority of the approaches based on Fuzzy Rough Sets for the interpretation of the phenomenon even if further experiments are needed, including, for example, those on big data which may require more efficient approaches such as the one reported in [28].

Further investigations will be carried out by validating the measurements with the most representative datasets of information disorder phenomena (such as stances, echo chambers, filter bubbles) and extending the measurements with techniques and methods, always based on the common framework of the Rough Set, for the analysis of communities [1] and diffusion networks [19]. In this direction, future developments will take into account two aspects: the information propagation model and methods that can help to understand the intentions of agents and interpreters. Regarding the first aspect, future developments will focus on the analysis of the results reported in [49] which include social factors (such as trust and motivation) and elements of game theory that are significant for particular phenomena of information disorder, such as propaganda in which the interpreters seem to attribute trust in undefined authorities and increase the propagation of mis-information, or even the echo chambers in which the interpreters appear to have benefits from the repetition of very similar points of view and opinions. Regarding the second point, the study will involve methods and techniques for the derivation and evaluation of hypotheses, such as those reported in [18] which are based on the same formal settings used in our work.

## Data Availability

The raw data elaborated during the current research are publicly available.

## Footnotes

1 https://www.who.int/docs/default-source/epi-win/infodemic-management/infodemiology-scientific-conference-booklet.pdf?sfvrsn=179de76a_4

2 https://www.politifact.com/

3 https://www.factcheck.org/

4 https://www.washingtonpost.com/news/fact-checker/

5 https://toolbox.google.com/factcheck/explorer

6 https://ifcncodeofprinciples.poynter.org/

7 $a_{\min \limits }$ and $a_{\max \limits }$ are respectively the minimum and maximum values assumed by *a*.

8 A closed system is a system that does not allow transfer of matter in or out of it.

9 In the Section 7, for the entropy values, 0 is used to indicate also the case in which a specific region has no parts. The text of the section explicitly mentions this situation.

10 Emotional valence refers to the pleasantness - higher values - or unpleasantness - lower values - of an emotional stimulus.

11 The package *syuzhet* https://cran.r-project.org/web/packages/syuzhet/vignettes/syuzhet-vignette.html

12 https://www.kaggle.com/kerneler/starter-covid-19-fake-news-dataset-19713c8a-1/datahttps://www.kaggle.com/kerneler/starter-covid-19-fake-news-dataset-19713c8a-1/data

13 https://developer.twitter.com/en/docs/api-reference-index

14 https://spacy.io/api

15 https://textblob.readthedocs.io/en/dev

16 https://cran.r-project.org/web/packages/RoughSets/index.html
